# A mirage under the water: underwater detection of an obscure anastomotic leakage

**DOI:** 10.1055/a-2794-7684

**Published:** 2026-02-24

**Authors:** Kengo Kasuga, Hiroomi Ogawa, Ayaki Isshiki, Shingo Ishihara, Takashige Masuo, Yoji Takeuchi, Toshio Uraoka

**Affiliations:** 1 37002 Department of Gastroenterology, Isesaki Municipal Hospital, Gunma, Japan; 2 Department of Gastroenterology and Hepatology, Gunma University Graduate School of Medicine, Gunma, Japan; 3 37002 Department of Surgery, Isesaki Municipal Hospital, Isesaki, Japan

The placement of an over-the-scope (OTS) clip is a useful treatment option for anastomotic leakage following gastrointestinal surgery 1 2 . Although OTS clips are effective in closing leaks smaller than 10 mm in diameter after colorectal surgery 3 , endoscopic identification of such small leaks can be challenging ( Video 1 ).

Underwater detection and endoscopic closure of an anastomotic leak following sigmoidectomy for colorectal cancer.Video 1

**Fig. 1:**
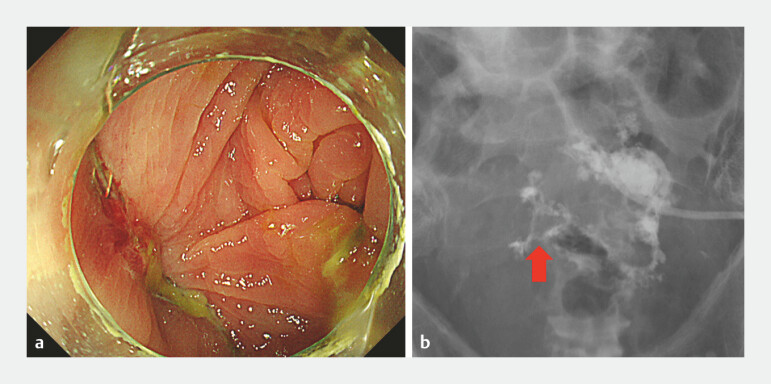
**a** No visible leakage site under CO₂ insufflation. **b** Contrast medium entering the colon from the intra-abdominal drain on fluoroscopy.

**Fig. 2:**
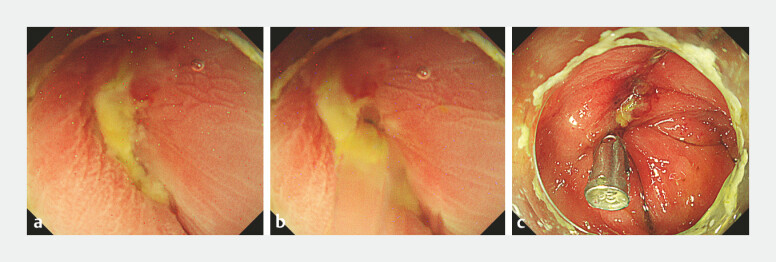
Endoscopic findings around the leakage site. **a** Before contrast injection via the abdominal drain. **b** Underwater view clearly showing turbid contrast entering the lumen. **c** Leak site closed using a through-the-scope (TTS) clip.

**Fig. 3:**
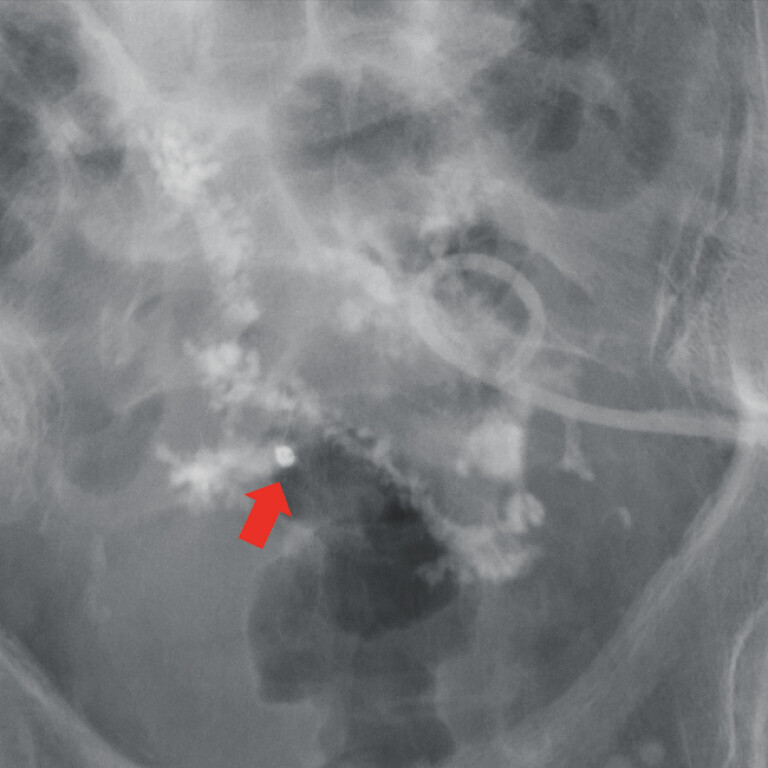
A fluoroscopic image after TTS clip placement; no further contrast inflow into the colon. TTS, through-the-scope.

**Fig. 4:**
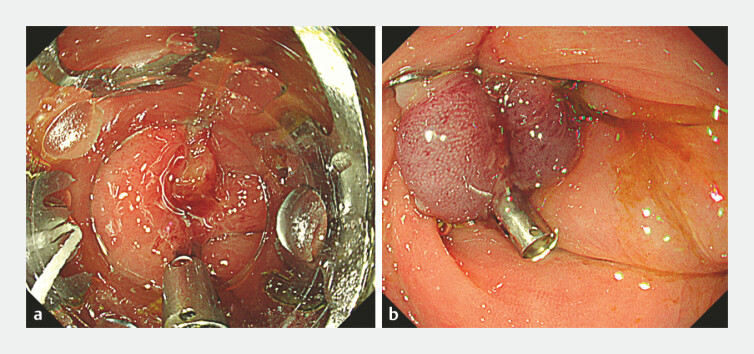
**a** Over-the-scope(OTS) clip just before deployment. **b** Complete closure of the leakage site after OTS clip placement.

**Fig. 5:**
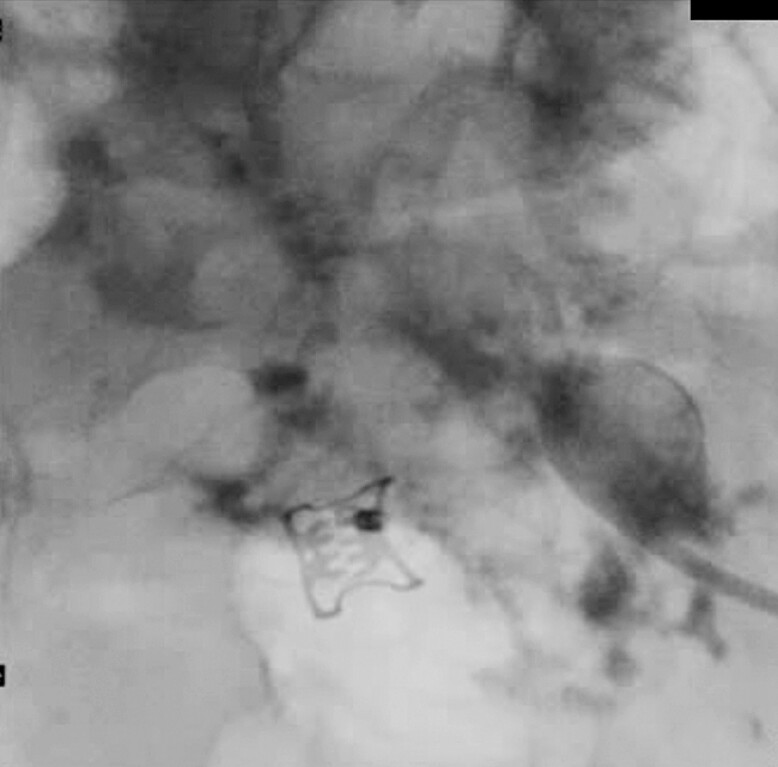
A fluoroscopic image after OTS clip placement; no further contrast inflow into the colon.

An 86-year-old man underwent sigmoidectomy for colon cancer. On postoperative day 2 (POD2), the anastomotic leakage was detected based on drainage fluid contamination. A drainage tube was placed under the computed tomographic guidance for an intra-abdominal abscess. On POD 16, the anastomotic site was examined via fluoroscopic colonoscopy. No leakage site was detected endoscopically; however, the contrast medium injected through the abdominal drain flowed into the colon ( Fig. 1 ). Under CO₂ insufflation, the exact entry point of the contrast medium could not be determined. However, underwater observation clearly visualized turbid contrast medium flow like a mirage under the water, allowing the precise localization of the leakage site. Subsequently, a through-the-scope (TTS) clip was applied ( Fig. 2 ). After clip placement, contrast medium flow ceased ( Fig. 3 ). Four days later, repeat fluoroscopy via an abdominal drain revealed residual leakage beside the clip. Consequently, a 9-mm OTS clip was placed ( Fig. 4 ). The procedure was completed using simple suction, and the previously placed clip did not interfere with the OTS clip placement. After the OTS clip placement, the contrast medium flow ceased ( Fig. 5 ). Fifteen days later, the patient was discharged without leakage recurrence.

Underwater endoscopy is a well-established technique that facilitates insertion and endoscopic resection during colonoscopy 4 5 . In the present case, underwater observation was valuable in detecting a small colonic anastomotic leak. Moreover, prior marking of the TTS clip did not interfere with OTS clip application. Our experience highlights the advantages of underwater endoscopy for identifying subtle anastomotic leaks.

Endoscopy_UCTN_Code_CCL_1AD_2AG Endoscopy_UCTN_Code_CPL_1AH_2AG Endoscopy_UCTN_Code_TTT_1AO_2AI
